# Validating attribute hierarchies in cognitive diagnosis models

**DOI:** 10.3389/fpsyg.2025.1562807

**Published:** 2025-04-28

**Authors:** Xueqin Zhang, Yu Jiang, Tao Xin, Yanlou Liu

**Affiliations:** ^1^Collaborative Innovation Center of Assessment for Basic Education Quality, Beijing Normal University, Beijing, China; ^2^Joint Logistics College, National Defense University, Beijing, China; ^3^China Academy of Education Big Data, Qufu Normal University, Qufu, China

**Keywords:** cognitive diagnosis model, attribute hierarchy, covariance matrix, information matrix, likelihood ratio test

## Abstract

Cognitive diagnosis models (CDMs) are restricted latent class models that are widely used in educational and psychological fields. Attribute hierarchy, as an important structural feature of the CDM, can provide critical information for inferring examinees’ attribute mastery patterns. Previous studies usually formulate likelihood ratio (LR) tests for full models and hierarchical models to validate attribute hierarchies, but their asymptotic distributions tend to become non-standard, resulting in test failures. This study proposes the Wald statistic to statistically test the *a priori* defined attribute hierarchy. Specifically, two covariance matrix estimators, empirical cross-product information matrix (XPD), and observed information matrix (Obs), are considered to compute the Wald statistic, referred to as Wald-XPD and Wald-Obs, respectively. Simulation studies with various factors were conducted to investigate the performance of the new methods. The results show that Wald-XPD has an acceptable empirical performance with high or low quality items and a higher test efficiency. Real datasets were also analyzed for illustrative purpose.

## 1 Introduction

Cognitive diagnosis models (CDMs) are essentially a popular class of multidimensional discrete latent variable models (Rupp et al., 2010) that model the relationship between observed variables and multidimensional latent variables, and are able to infer fine-grained information about examinees’ mastery or non-mastery of a set of attributes according to their observed item responses. Theoretical studies on CDM have been of great interest to researchers in recent decades. The three most widely used saturated CDMs are the generalized deterministic input, noisy “AND” gate (G-DINA) model (de la Torre, 2011), the log-linear CDM (LCDM; Henson et al., 2009), and the general diagnostic model (GDM; von Davier, 2008), respectively. Some special cases of saturated CDMs can be obtained under specific constraints, such as the deterministic input, noisy “AND” gate model (DINA; Haertel, 1989), the deterministic input, noisy output “OR” gate model (DINO; Templin and Henson, 2006).

With the widespread application of CDM in different disciplines, such as education, psychology, and medicine, the term “attributes” has taken on different scientific meanings, which can be knowledge or skills acquired by students (de la Torre, 2011; Junker and Sijtsma, 2001), characteristics of specific psychological disorders (de la Torre et al., 2018; Templin and Henson, 2006), or pathogen characteristics of specific diseases (Wu et al., 2017). In methodological studies of CDM, latent attributes are typically assumed to be sequentially ordered (Liu, 2018; Templin and Bradshaw, 2014), implying that mastery of attributes is also a progressive process, with mastery of lower-level attributes usually being a prerequisite for mastery of higher-level attributes (Jimoyiannis and Komis, 2001; Leighton et al., 2004; Liu, 2018; Templin and Bradshaw, 2014; Wang and Gierl, 2011). This dependency between attributes has been formalized as an “attribute hierarchy” (Leighton et al., 2004) or “attribute structure” (Liu and Huggins-Manley, 2016; Liu et al., 2017). Leighton et al. (2004) originally introduced the term “attribute hierarchy” to facilitate the description of prerequisite relationships between latent attributes in different domains and proposed four different types of attribute hierarchies: linear, convergent, divergent, and unstructured.

The attribute hierarchy can reveal an examinee’s mental processing of learning a set of latent knowledge or skills, provide meaningful guidance to the cognitive diagnostic assessment in designing test items and analyzing data, and generate guidance recommendations or remediation strategies accordingly (Ma et al., 2023). However, accurately defining the attribute hierarchy is a very challenging task, and most of the applied studies did not likely use a hierarchy (Sessoms and Henson, 2018). Previous research has shown that misspecification of the attribute hierarchy directly affects the model-data fit at the item level as well as the classification accuracy (Liu et al., 2017; Liu, 2018). Therefore, any prespecified attribute hierarchy should be supported by theoretical constructs or data (Bradshaw et al., 2014; Liu, 2018; Templin and Bradshaw, 2014).

Recently, attribute hierarchies in CDM have attracted increasing research interest. Gierl et al. (2007) was the first to propose the attribute hierarchy method (AHM) for diagnosing examinees’ attribute mastery patterns, which explicitly defines an *a priori* attribute hierarchy and classifies the examinees into predefined structured attribute mastery patterns. However, AHM emphasizes the construction of attribute hierarchies according to the theory of construction in the adjacency matrix, it lacks inferential statistics to validate the a priori assumptions of the attribute hierarchy (Templin and Bradshaw, 2014). Templin and Bradshaw et al. (2014) considered attribute hierarchies in LCDM and proposed the hierarchical diagnostic classification model (HDCM), which restricts impermissible structural and item parameters in saturated LCDM to zero. Then, they formulated a LR test on the saturated model and the HCDM to validate the existence of a prespecified attribute hierarchy. Similarly, Hu and Templin (2020) performed an LR test on Bayesian inference networks based on a model comparison framework to validate the existence of a prespecified attribute hierarchy in Bayesian inference network models. Researchers (Ma and Xu, 2021; Wang and Lu, 2021) found that the asymptotic distribution of the LR test becomes non-standard, and this non-standard limiting distribution is very slow to converge, and even prone to test failure. Ma and Xu (2021) further proposed resampling-based (e.g., parametric and nonparametric resampling) LR to validate attribute hierarchies in CDM. Although the resampling-based approach avoids the series of problems of the traditional LR test, a general problem with the approach is the high computational and time costs, especially for large-scale datasets. In addition, when the attribute hierarchy is unknown, researchers have proposed several exploratory methods to infer the attribute hierarchy. For example, Wang and Lu (2021) proposed to learn the attribute hierarchy from the data using a latent variable selection method (Xu and Shang, 2018) and a regularized latent class modeling method (Chen et al., 2017). Liu et al. (2022) introduced a z-test based on the structural parameter standard errors (SEs) to explore the attribute hierarchy. However, the performance of this method is more sensitive to the structural parameter estimation. Zhang et al. (2024) further proposed an iterative method for exploring attribute hierarchies, aiming to solve the problem associated with the instability of standard error estimation. However, the problem of subjectivity in setting thresholds during the iterative process remains unresolved. When the goal is to explore an unknown attribute hierarchy, the exploratory approach described above is more appropriate. In contrast, this study focuses on the statistical testing of attribute hierarchies and aims to develop a statistical method to validate pre-specified attribute hierarchies that is not data-driven, and also avoids the problems associated with irregular standard error estimation and subjectivity associated with threshold setting.

The attribute hierarchy can be directly reflected in the set of latent attribute mastery patterns. Under the constraint of the attribute hierarchy, the number of attribute mastery patterns is much less than $2^{K}$ because some attribute mastery patterns are impermissible (Liu et al., 2022), that is, the structural parameter estimates corresponding to these impermissible attribute mastery patterns should not be significantly larger than 0 in the saturated CDM. Validating the attribute hierarchy can essentially be equivalent to testing the significance of a specific set of structural model parameter estimates under the attribute hierarchy constraint. If a set of impermissible structural parameter estimates specified by an attribute hierarchy is not significantly different from 0, then statistical evidence can be provided to support the existence of an attribute hierarchy. Consequently, if a specific attribute hierarchy is assumed, the Wald statistic can be used to validate the existence of the attribute hierarchy.

The calculation of the Wald statistic is based on the covariance matrix of the model parameter estimates, and the accuracy of the covariance matrix estimation has a significant impact on the Wald test. The covariance matrix of the model parameters can be obtained by inverting the information matrix. In CDM, researchers have proposed a variety of information matrix estimation methods for complete data (Liu et al., 2019b; Philipp et al., 2018), such as empirical cross-product (XPD) matrix, observed information (Obs) matrix (Louis, 1982), and sandwich-type covariance (Sw) matrix (Huber, 1967), and these methods have important application value in the fields of model parameter’s standard error estimation (Philipp et al., 2018; Liu et al., 2022), attribute hierarchy testing (Liu et al., 2022), differential item functioning detection (Hou et al., 2014; Liu et al., 2019a,b; Ma et al., 2017), and model comparison (Liu et al., 2019a; Ma and de la Torre, 2019; Ma et al., 2016; de la Torre, 2011). For example, Philipp et al. (2018) evaluated the performance of standard error estimation for complete versus non-complete XPD matrices, and found that complete information matrices provide more accurate SE estimation. Liu et al. (2019b) systematically evaluated the performance of covariance matrix estimators based on complete Obs and Sw in the LCDM framework and found that, with a correctly specified model, both methods have good performance.

The main goal of this study is to propose a new method for validating attribute hierarchies based on the Wald statistic. It is not clear how well the available covariance estimators perform with the Wald statistic used for attribute hierarchy testing. Considering that the computation of the Wald statistic relies on the covariance of the structural parameter estimates, and the Sw covariance estimator suffers from estimation bias in the SE estimates of the structural parameters (Liu et al., 2022). Therefore, as a preliminary attempt, the Wald test in this study considers the Obs and XPD matrices, which are two covariance matrix estimators that have been widely discussed and used in the existing literature, and their selection will help in comparing and validating with the existing studies. For ease of presentation, these two statistics are referred to as Wald-XPD and Wald-Obs, respectively. Then, two simulation studies were conducted to evaluate the empirical performance of Wald-XPD, Wald-Obs and LR statistics for validating attribute hierarchies in CDM. It is known that different covariance matrix estimators have different computational forms and do not perform exactly the same in the inferential statistics applications mentioned above, and one can anticipate that the two Wald tests may present different performances under specific conditions. Although the LR test is not affected by the estimation of the variance matrix, it tends to fail when the number of items is large (Ma and Xu, 2021), and whether the Wald statistic has certain advantages remains to be further explored.

The rest of the paper is organized as follows: First, the theory of HDCM and LR testing is briefly reviewed. Second, the newly developed attribute hierarchy validation methods are described in detail. Third, two simulation studies were conducted to evaluate and compare the performance of various attribute hierarchy validation methods in terms of empirical Type I error control rate and statistical power under different simulation conditions, and the attribute hierarchies of a common empirical dataset are analyzed. Finally, the study results were discussed and summarized.

## 2 Method

### 2.1 Hierarchical diagnostic classification models

Log-linear models with latent variables use latent class analysis to model the relationships between categorical variables and can easily be generalized to obtain CDMs (von Davier, 2008; Henson et al., 2009). The LCDM is a representative log-linear model, which defines attribute main effects and interaction effects. The LCDM can be simplified into other constrained CDMs by applying different constraints to its parameters (Henson et al., 2009), as described below. The item response function of the LCDM is expressed as Equation (1):


(1) $$P\left(x_{ij}=1|\alpha_{i}\right)=\frac{\exp\left(\lambda_{j,0}+\lambda_{j}^{T}h\left(\alpha_{i},q_{j}\right)\right)}{1+\exp\left(\lambda_{j,0}+\lambda_{j}^{T}h\left(\alpha_{i},q_{j}\right)\right)}$$

where $x_{ij}$ is the response of examinee *i* with attribute mastery pattern $\alpha_{i}$ to item $j\in \left\{1,2\ldots ,J\right\}$ , $q_{j}$ is a row vector of a binary J×K matrix Q , and *K* is the number of attributes. For item *j*, $q_{j}=\left(q_{j1},\ldots ,q_{jk},\ldots ,q_{jK}\right)$ , if the *k*th attribute is required for item *j*, then $q_{jk}=1$ ; otherwise, $q_{jk}=0$ . Moreover, $\lambda_{j,0}$ denotes an intercept parameter, and $\lambda_{j}^{T}h\left(\alpha ,i,q_{j}\right)$ denotes the main effects and interaction effects between attributes, which have a size of $2^{K}-1$ . $\lambda_{j}^{T}h\left(\alpha_{i},q_{j}\right)$ is expressed as Equation (2):


(2) $$\begin{array}{l} \lambda_{j}^{T}h\left(\alpha_{i},q_{j}\right)=\overset{K}{\underset{k=1}{\sum }}\lambda_{j,k}\alpha_{ik}q_{jk}\\ +\overset{K}{\underset{k=1}{\sum }}\;\underset{k'>k}{\sum }\lambda_{j,kk'}\alpha_{ik}\alpha_{ik'}q_{jk}q_{jk'}+\ldots +\lambda_{j,12\ldots K}\overset{K}{\underset{k=1}{\sum }}\alpha_{ik}q_{jk} \end{array}$$

where $\lambda_{j,k}$ is the main effect caused by $\alpha_{k}$ , $\lambda_{j,kk'}$ is the two-way interaction effect between $\alpha_{k}$ and $\alpha_{k'}$ , $\lambda_{j,12\ldots K}$ is the *K*-way interaction effect. In addition, the full LCDM includes a structural parameter vector $\pi ={\left(\pi_{1},\ldots ,\pi_{l},\ldots ,\pi_{L}\right)}^{'}$ , where $\pi_{l}$ describes the probability of a randomly selected examinee belonging to the *l*th attribute mastery pattern. The LCDM assumes that an examinee’s attribute mastery pattern can be one of the $L=2^{K}$ possible attribute mastery patterns and that $\overset{L}{\underset{l=1}{\sum }}\pi_{l}=1$ . Therefore, in addition to the item parameters, the full LCDM includes $2^{K}-1$ structural parameters that should be estimated.

Suppose that the *q* vector of item *j* is $q_{j}=\left(1,1\right)$ , where attribute $\alpha_{1}$ is a prerequisite for attribute $\alpha_{2}$ . In the LCDM, the probability that examinee *i* correctly answers item *j* is expressed as Equation (3):


(3) $$P\left(x_{ij}=1|\alpha_{i}\right)=\frac{\exp\left(\lambda_{j,0}+\lambda_{j,1}\alpha_{i1}+\lambda_{j,2}\alpha_{i2}+\lambda_{j,12}\alpha_{i1}\alpha_{i2}\right)}{1+\exp\left(\lambda_{j,0}+\lambda_{j,1}\alpha_{i1}+\lambda_{j,2}\alpha_{i2}+\lambda_{j,12}\alpha_{i1}\alpha_{i2}\right)}$$

there are four item parameters should be estimated, and three structural parameters must be estimated because $\overset{L}{\underset{l=1}{\sum }}\pi_{l}=1$ . The model parameters of the LCDM are redundant if an attribute hierarchy exists, Templin and Bradshaw et al. (2014) proposed an HDCM model to accommodate the attribute hierarchy, this model is nested within the full LCDM, in which some redundant parameters are set to 0 to simplify the parameters. The item response function of an HDCM is expressed as Equation (4),


(4) $$P\left(x_{ij}=1|\alpha_{i}\right)=\frac{\exp\left(\lambda_{j,0}+\lambda_{j,1}\alpha_{i1}+\lambda_{j,12}\alpha_{i1}\alpha_{i2}\right)}{1+\exp\left(\lambda_{j,0}+\lambda_{j,1}\alpha_{i1}+\lambda_{j,12}\alpha_{i1}\alpha_{i2}\right)}$$

where the main effect of attribute $\alpha_{2}$ is removed because $\alpha_{2}$ is nested in $\alpha_{1}$ , and the structural parameters are simplified. In HCDM, Templin and Bradshaw (2014) proposed the LR test to validate the predetermined attribute hierarchy. The LR test can be written as Equation (5)


(5) $$LR=-2\log\left[\frac{L_{r}\left(\tilde{\gamma }\right)}{L_{s}\left(\hat{\gamma }\right)}\right]=2\left[{𝓁}_{s}\left(\hat{\gamma }\right)-{𝓁}_{r}\left(\tilde{\gamma }\right)\right]$$

where $L_{r}\left(\tilde{\gamma }\right)$ represents the likelihood value for an HDCM and $L_{s}\left(\hat{\gamma }\right)$ represents the likelihood values for the corresponding saturated CDM. Under the null hypothesis, the HDCM with attribute hierarchy is the “true” model, and the statistics asymptotically follow a Chi-Square distribution with number of degrees of freedom equal to the difference in the number of free parameters between the saturated CDM and the reduced CDM (Templin and Bradshaw, 2014).

### 2.2 The Wald statistic for testing attribute hierarchy

If an attribute hierarchy exists, there are some structural parameters that are impermissible when the saturated CDM is used to fit the observed response data, which have true values of 0 and estimates that are very close to, or even equal to zero. Therefore, significance tests of the estimated structural parameters can provide statistical evidence in favor of the pre-specified attribute hierarchy.

In this section, we illustrate how the Wald statistic can be used to validate the attribute hierarchy. Specifically, the procedure for validating the attribute hierarchy in the CDM by using the Wald statistic is as follows: first, a saturated CDM was used to fit the examinee’s observed response data, and the item and structural parameter vectors of the model are estimated using the MMLE-EM algorithm. Then, the set of structural parameter vectors to be tested is determined based on the pre-specified attribute hierarchy. The focus of this step is to construct the constraint matrix R based on the attribute hierarchy to be validated. To illustrate with a specific example, suppose item *j* measures three attributes and the q vector is $q_{j}=\left(1,1,1\right)$ . For a saturated CDM, the number of possible attribute mastery patterns is and the structural parameter vector to be estimated is $\hat{\pi }=\left(\pi_{1},\pi_{2},\pi_{3},\pi_{4},\pi_{5},\pi_{6},\pi_{7}\right)$ . If there exists a linear hierarchical relationship between attributes, that is, attribute $\alpha_{1}$ is a prerequisite for attribute $\alpha_{2}$ and attribute $\alpha_{2}$ is a prerequisite for attribute $\alpha_{3}$ . The attribute mastery pattern $\alpha_{3}\left(0,1,0\right)$ , $\alpha_{4}\left(0,0,1\right)$ , $\alpha_{6}\left(1,0,1\right)$ and $\alpha_{7}\left(0,1,1\right)$ are impermissible, that is the structural parameters $\pi_{3}$ , $\pi_{4}$ , $\pi_{6}$ , and $\pi_{7}$ should not be significantly larger than zero. Validating the existence of an attribute hierarchy is equivalent to testing whether the structural parameters $\pi_{3}$ , $\pi_{4}$ , $\pi_{6}$ , and $\pi_{7}$ are simultaneously significantly larger than zero. Specifically, the following constraint matrix R can be constructed as shown in Equation (6).


(6) $$R=\left(\begin{array}{llllllll} 0 & 0 & 1 & 0 & 0 & 0 & 0 & 0\\ 0 & 0 & 0 & 1 & 0 & 0 & 0 & 0\\ 0 & 0 & 0 & 0 & 0 & 1 & 0 & 0\\ 0 & 0 & 0 & 0 & 0 & 0 & 1 & 0 \end{array}\right)$$

The vector of structural parameters to be tested is then obtained by matrix multiplication $R\hat{\pi }$ as shown in as Equation (7),


(7) $$R\hat{\pi }=\left({\hat{\pi }}_{3},{\hat{\pi }}_{4},{\hat{\pi }}_{6},{\hat{\pi }}_{7}\right)$$

Subsequently, the Wald statistic for the attribute hierarchy test can be expressed as Equation (8),


(8) $$\mathrm{Wald}={\left(R\hat{\pi }\right)}^{\prime }{\left(R{\hat{\Sigma }}_{\left(\pi ,\pi \right)}R^{\prime }\right)}^{-1}\left(R\hat{\pi }\right)$$

where ${\hat{\Sigma }}^{\left(\pi ,\pi \right)}$ is the covariance matrix of the estimated structural parameters. This covariance matrix is expressed as Equation (9):


(9) $${\hat{\Sigma }}^{\left(\pi ,\pi \right)}=\left[\begin{array}{ccc} {\hat{\Sigma }}^{\left(\pi_{1},\pi_{1}\right)} & \ldots & {\hat{\Sigma }}^{\left(\pi_{1},\pi_{L-1}\right)}\\ \vdots & \ddots & \vdots \\ {\hat{\Sigma }}^{\left(\pi_{L-1},\pi_{1}\right)} & \cdots & {\hat{\Sigma }}^{\left(\pi_{L-1},\pi_{L-1}\right)} \end{array}\right]$$

The accuracy of the estimated covariance matrix ${\hat{\Sigma }}^{\left(\pi ,\pi \right)}$ has a significant impact on the performance of the Wald statistic. In this study, two methods of information matrix estimation for observed data proposed by Liu et al. (2019b) and Philipp et al. (2018) are used to obtain the covariance matrices of the structural parameters, which are XPD and Obs matrix. Specifically, the XPD matrix is obtained by taking the cross-product of the derivative of the log-likelihood function of the observed data with respect to the model parameter vector $\gamma =\left(\lambda ,\pi \right)$ . This matrix is defined in Equation (10),


(10) $$I_{XPD}=\left[\begin{array}{ccc} \frac{\partial 𝓁\left(\hat{\gamma }|x\right)}{\partial \lambda_{1}}\frac{\partial 𝓁\left(\hat{\gamma }|x\right)}{\partial \lambda_{1}} & \ldots & \frac{\partial 𝓁\left(\hat{\gamma }|x\right)}{\partial \lambda_{1}}\frac{\partial 𝓁\left(\hat{\gamma }|x\right)}{\partial \pi_{L-1}}\\ \vdots & \ddots & \vdots \\ \frac{\partial 𝓁\left(\hat{\gamma }|x\right)}{\partial \pi_{L-1}}\frac{\partial 𝓁\left(\hat{\gamma }|x\right)}{\partial \lambda_{1}} & \cdots & \frac{\partial 𝓁\left(\hat{\gamma }|x\right)}{\partial \pi_{L-1}}\frac{\partial 𝓁\left(\hat{\gamma }|x\right)}{\partial \pi_{L-1}} \end{array}\right]$$

The Obs matrix is the negative second derivative of the log-likelihood function of the observed data matrix with respect to the model parameters. This matrix is expressed in Equation (11),


(11) $$I_{Obs}=-\left[\begin{array}{ccc} \frac{\partial^{2}𝓁\left(\hat{\gamma }|x\right)}{\partial \lambda_{1}\partial \lambda_{1}} & \ldots & \frac{\partial^{2}𝓁\left(\hat{\gamma }|x\right)}{\partial \lambda_{1}\partial \pi_{L-1}}\\ \vdots & \ddots & \vdots \\ \frac{\partial^{2}𝓁\left(\hat{\gamma }|x\right)}{\partial \pi_{L-1}\partial \lambda_{1}} & \cdots & \frac{\partial^{2}𝓁\left(\hat{\gamma }|x\right)}{\partial \pi_{L-1}\partial \pi_{L-1}} \end{array}\right]$$

The covariance matrix of the model parameters can be obtained by inverting the information matrix (Liu et al., 2019a,b; Philipp et al., 2018). Similar to the information matrix, the covariance matrix consists of four block matrices and can be written as Equation (12),


(12) $$\Sigma_{XPD}=\left[\begin{array}{cc} \Sigma_{XPD}^{\left(\lambda ,\lambda \right)} & \Sigma_{XPD}^{\left(\lambda ,\pi \right)}\\ \Sigma_{XPD}^{\left(\pi ,\lambda \right)} & \Sigma_{XPD}^{\left(\pi ,\pi \right)} \end{array}\right]$$

where $\Sigma_{XPD}^{\left(\lambda ,\lambda \right)}$ is the covariance matrix of the item parameter and $\Sigma XPD\left(\pi ,\pi \right)$ is the covariance matrix of the structural parameter. The Wald statistic calculated using the XPD and Obs matrices for the attribute hierarchy test is denoted as Wald-XPD and Wald-Obs, respectively.

Finally, a significance test is performed based on the values of the Wald statistics. In the Wald test, the null hypothesis H0 is “the value of the set of structural parameters to be tested is equal to 0” and the alternative hypothesis is “the value of the set of structural parameters to be tested is significantly larger than 0.” If fails to reject the null hypothesis at the pre-specified level of significance, then the Wald statistics for the structural model parameters will provide strong evidence to support the existence of the hypothesized attribute hierarchy.

## 3 Simulation studies

Two simulation studies were conducted to systematically evaluate the empirical performance of the Wald statistic computed by two covariance matrix estimators for validating attribute hierarchies and to compare it with the LR statistic under different conditions. Study 1 investigated the empirical Type I error control rate of these statistics when testing *a priori* defined attribute hierarchy, and Study 2 examined the statistical power of these statistics when there are no attribute hierarchies in the data.

### 3.1 Simulation study 1

#### 3.1.1 Design

Several factors that may affect the performance of attribute hierarchy testing are manipulated here, including sample size (*N*), number of attributes (*K*), item quality (*IQ*), type of attribute hierarchy, attribute distribution, and attribute hierarchy validation method. In this case, the attribute hierarchy validation verification method is a within-subjects factor and the other factors are between-subjects factors. Specifically, three different sample sizes were considered: *N* = 200, *N* = 500, or *N* = 1,000 for small, medium, and large sample sizes, respectively. This setting has been widely used in previous CDM methodology studies (e.g., Ma et al., 2016; Sen and Cohen, 2021; Sun et al., 2024). The number of attributes was *K* = 3 and *K* = 5, which is a more common design in simulation or real data studies of CDM (e.g., Sen and Cohen, 2021; Templin and Bradshaw, 2014). According to Liu and Huggins-Manley (2016) and Liu (2018) on attribute hierarch. When *K* = 3, there are three levels of types of attribute hierarchy structure: linear, pyramidal, and inverted pyramid (Liu, 2018). When *K* = 5, there are four types of attribute hierarchical structures: linear, pyramidal, inverted pyramid and diamond. Some researchers (e.g., de la Torre, 2009; Leighton et al., 2004; Liu and Huggins-Manley, 2016; Liu, 2018), use directed acyclic graphs and binary vectors to visualize the external shape and internal organization of attribute hierarchies. In this context, the internal organization refers to the assumed permissible and impermissible attribute mastery patterns, which are represented by 0–1 vectors. The different types of attribute hierarchies are listed in Table 1. Three levels of item quality were considered here: high, medium, and low quality, respectively. This setting has been widely used in previous studies (e.g., Ma and de la Torre, 2016, 2019; Ma and Xu, 2021; Sorrel et al., 2017). Item quality was defined by the parameters $P_{j}\left(0\right)$ and $P_{j}\left(1\right)$ , respectively. For all items, $P_{j}\left(0\right)$ represents the correct response probabilities of individuals who possesses none of the required attributes, and is fixed at $P_{j}\left(0\right)=0.1$ , 0.2, or 0.3 for high, medium, and low item quality, respectively. $P_{j}\left(1\right)$ represents the correct response probabilities of individuals who master all of the required attributes, and is fixed at $P_{j}\left(1\right)=0.9$ , 0.8, or 0.7 for high, moderate, and low item quality, respectively. The attribute mastery patterns followed two distributions: uniform and non-uniform distribution. For the uniform distribution, all attribute mastery pattern is randomly generated from the permissible attribute mastery patterns with an equal probability. For non-uniform distributions, the design of Liu et al. (2022) was used here to obtain attribute mastery patterns. Specifically, the examinee’s attribute mastery pattern was generated from a dichotomized multivariate normal distribution whose mean vector was set to 0, and the off-diagonal elements of the covariance matrix were randomly drawn from the uniform distribution $\mu \left(0.5,0.8\right)$ . In addition, three attribute hierarchy validation methods were used in this study, including the Wald test based on two covariance matrix estimators (Wald-XPD and Wald-Obs), and the LR statistic.

**Table 1:** External shape and internal organization of attribute hierarchy.

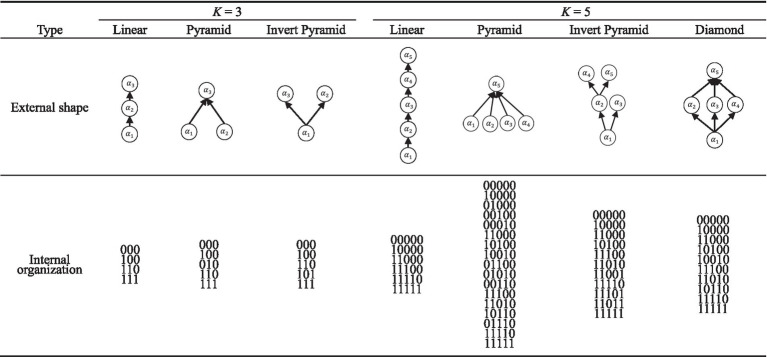

#### 3.1.2 Data generation

**Figure 1:**
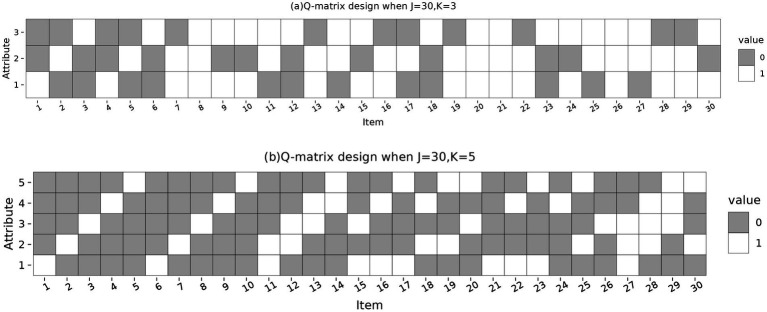
*Q*-matrix design for the simulated data. (J): Number of items, (K): Number of attributes, (Value: 0): Attribute not measured, (Value: 1): Attribute measured.

The data generation model was the HDCM with the identity link function. The process of data generation is as follows: First, generate the attribute mastery patterns of the examinees based on uniform or non-uniform distributions. Second, generate the *Q*-matrix and item parameters. Specifically, the test length for the entire simulation experiment was set to 30 items, and the attributes measured for each item are shown in the *Q* matrix in Figure 1, the *Q* matrix contained two unit submatrices, and the remaining items were randomly generated. The *Q* matrix used here satisfies the identifiability conditions proposed by Gu and Xu (2020) for CDMs in general or with attribute hierarchies. Similar to Liu et al. (2022), the main and interaction effects for each item were set to the same value equal to $P\left(1\right)-P\left(0\right)/s_{j}$ , $s_{j}$ being the number of main and interaction effects required for item *j*. Then, item response data were randomly generated based on the HDCM. Specifically, the probability of a correct response was calculated using the HDCM and compared to a random number ranging from 0 to 1. If the item response probability was greater than the random number, the item response was coded as 1, and vice versa the response was 0 (Sun et al., 2024). Finally, the G-DINA model with an identity link function is used to fit the response data.

In all conditions, each experiment was repeated 500 times in order to obtain stable estimates. In each case, the percentage of replicates where H_0_ was rejected was observed. All simulation experiment codes were written in R software. The GDINA R package (Ma and de la Torre, 2020) was used to estimate model parameters. The R code for estimating the covariance matrix of the structural parameters by using Obs and XPD matrices was modified from Liu et al. (2022).

#### 3.1.3 Evaluation criteria

The empirical Type I errors rate was used as an evaluation criteria. Type I errors occur when a hypothesis test concludes that an attribute hierarchy does not exist in the data, in fact an attribute hierarchy exists in the data. In each condition, the empirical Type I error rate is the percentage of times that the hypothesis test makes the Type I error in *n* replications at a specific significance level. Due to sampling error. The Type I error rate may not be exactly equal to the pre-specified significance level. When the researcher chose a significance level of 0.05 with *n* replications for each condition, the 95% confidence interval for the observed Type I error rate can be expressed as $p\pm 1.96\sqrt{p\left(1-p\right)/n}$ , which means that there is a 95% chance that the observed Type I error rate will fall within the interval [0.031, 0.069].

#### 3.1.4 Results

Table 2 presents the average empirical Type I error rates for the three methods under various conditions for high-quality items when K = 3. As shown, all three methods generally exhibit conservative Type I empirical error rates in most conditions for high quality items. Specifically, the Wald-XPD method yields an empirical type I error rate that is very close to zero, but not quite zero, and the Wald-Obs method performs similarly, with an empirical type I error rate close to the nominal level of 0.05 only when testing linear attribute hierarchies with *N* = 500 and 1,000. In contrast, the LR test is consistently overly conservative for high quality items, with Type I error rates equal of zero in all conditions, regardless of sample size, attribute hierarchy, or population distribution.

**Table 2:** The empirical Type I error rates for Wald-XPD, Wald-Obs, and LR when *K* = 3 and items were of high quality ( α=0.05 ).

		Uniform			Non-uniform		
Structure	*N*	W-XPD	W-Obs	LR	W-XPD	W-Obs	LR
Linear	200	0.004	0.012	0	0.006	0.008	0
	500	0.002	**0.043**	0	0.008	0.027	0
	1,000	0	**0.056**	0	0.002	0.071	0
Inverted pyramid	200	0.02	0	0	0.004	0.006	0
	500	0.006	0.004	0	0.002	0.006	0
	1,000	0.004	0.001	0	0.004	0.004	0
Pyramid	200	0.012	0.002	0	0	0.000	0
	500	0.008	0.008	0	0.01	0.004	0
	1,000	0.008	0.004	0	0.008	0.006	0

Structure represents attribute hierarchy type; *N* is the sample size. LR is the likelihood ratio test; W-Obs is the Wald statistic calculated using the observed information matrix; W-XPD is the Wald statistic calculated using the empirical cross-product matrix; Uniform is the uniform population distribution; non-uniform is the non-uniform population distribution. Linear is a linear hierarchy; Inverted pyramid is an inverted pyramid hierarchy; pyramid is a pyramid hierarchy. The bold font represents an acceptable empirical Type I error rates at a significance level of 0.05.

Table 3 presents the average empirical Type I error rates of the three methods under different conditions for moderate quality items when *K* = 3. Several observations can be made: First, as item quality decreases, the Wald-XPD statistic becomes more conservative for moderate-quality items, with empirical Type I error rates consistently zero across all conditions. Second, the Wald-Obs statistic exhibits inflated empirical Type I error rates for moderate-quality items, with rates exceeding the nominal level in all conditions. Finally, as item quality decreases, the empirical Type I error rates of the LR statistic increase under certain conditions. For instance, the LR statistic produces better Type I error rates under the linear attribute hierarchies at *N* = 200, and under the Pyramid structure at *N* = 1,000, but remained more conservative under the other conditions.

**Table 3:** The empirical Type I error rates for Wald-XPD, Wald-Obs, and LR when *K* = 3, and items of moderate quality ( α=0.05 ).

		Uniform			Non-uniform		
Structure	*N*	W-XPD	W-Obs	LR	W-XPD	W-Obs	LR
Linear	200	0	0.312	**0.042**	0	0.265	**0.05**
	500	0	0.39	0.072	0	0.372	0.076
	1,000	0	0.488	0.106	0	0.46	0.108
Inverted pyramid	200	0	**0.057**	0.004	0	0.145	0.022
	500	0	0.093	0.014	0	0.125	0.024
	1,000	0	0.119	0.02	0	0.18	0.016
Pyramid	200	0	0.085	0.016	0	0.112	0.016
	500	0	0.089	0.024	0	0.128	0.014
	1,000	0	0.312	**0.042**	0	0.265	**0.05**

The bold font represents an acceptable empirical Type I error rates at a significance level of 0.05.

Table 4 presents the average empirical Type I error rates of the three methods under different conditions with low-quality items when *K* = 3. As item quality decreases, the Wald-Obs statistic produces increasingly inflated empirical Type I error rates. Similarly, the LR statistic also produces inflated empirical Type I error rates across all conditions. In contrast, only the Wald-XPD statistic performs relatively well. For instance, when testing the linear attribute hierarchy under both population distributions, the Wald-XPD statistic achieves empirical Type I error rates near the nominal level of 0.05, but remains conservative under other conditions.

**Table 4:** The empirical Type I error rates for Wald-XPD, Wald-Obs, and LR when *K* = 3, and items of low quality ( α=0.05 ).

		Uniform			Non-uniform		
Structure	*N*	W-XPD	W-Obs	LR	W-XPD	W-Obs	LR
Linear	200	**0.056**	0.856	0.632	**0.064**	0.882	0.626
	500	**0.064**	0.801	0.63	**0.06**	0.799	0.628
	1,000	**0.038**	0.783	0.666	**0.04**	0.762	0.712
Inverted pyramid	200	0.016	0.761	0.454	**0.046**	0.769	0.494
	500	0.006	0.632	0.288	**0.054**	0.714	0.426
	1,000	0.002	0.574	0.264	**0.034**	0.676	0.386
Pyramid	200	0.018	0.757	0.432	0.026	0.771	0.454
	500	0.01	0.653	0.358	**0.032**	0.681	0.378
	1,000	0.014	0.57	0.302	0.018	0.661	0.372

The bold font represents an acceptable empirical Type I error rates at a significance level of 0.05.

Table 5 presents the average empirical Type I error rates of the three methods under different conditions for high-quality items when *K* = 5. It can be seen that the empirical performance of both the Wald-XPD and Wald-Obs statistics improve as the number of attributes increases. The Wald-XPD statistic performs well when testing the pyramid, inverted pyramid, and diamond attribute hierarchies under the uniform population distribution condition when *N* = 200, yielding empirical Type I error rates close to the nominal level of 0.05, although it remains more conservative in other conditions. Under a uniform distribution, the Wald-Obs statistic produces an empirical Type I error rate close to 0.05 when testing the pyramid structure. Under non-uniform population distribution, the performance of both the Wald-XPD and Wald-Obs statistics is similar to that of the uniform distribution. In contrast, as the number of attributes increases, the LR statistic remains overly conservative under high quality items, and its empirical Type I error rates are consistently zero under all conditions, regardless of sample size, attribute hierarchy structure, or population distribution.

**Table 5:** The empirical Type I error rates for Wald-XPD, Wald-Obs, and LR when *K* = 5 and items were of high quality ( α=0.05 ).

		Uniform			Non-uniform		
Structure	*N*	W-XPD	W-Obs	LR	W-XPD	W-Obs	LR
Linear	200	0.094	0.007	0	0.088	0.011	0
	500	**0.032**	0.025	0	**0.036**	0.025	0
	1,000	0.018	0.03	0	0.016	**0.04**	0
Inverted pyramid	200	0.07	0.013	0	0.026	0.007	0
	500	0.014	0.014	0	0.014	0.014	0
	1,000	0.01	0.026	0	0.004	0.012	0
Pyramid	200	**0.044**	0.035	0.006	**0.058**	0.009	0.002
	500	0.026	**0.039**	0.002	0.016	0.026	0
	1,000	0.006	**0.037**	0	0.004	0.014	0.002
Diamond	200	**0.056**	0.027	0.002	**0.066**	0.009	0
	500	0.026	0.021	0	**0.032**	0.018	0
	1,000	0.008	0.025	0	0.006	0.027	0

The bold font represents an acceptable empirical Type I error rates at a significance level of 0.05.

Table 6 presents the average empirical Type I error rates of the three methods under different conditions for moderate-quality items when *K* = 5. It is evident that as item quality decreases, the Wald-XPD statistic becomes more conservative under both population distributions, with its empirical Type I error rate approaching zero in most cases. The LR statistic shows some improvement under certain conditions, such as with the pyramid structure at *N* = 1,000, where the empirical Type I error rate produced by the LR statistic is close to the nominal level of 0.05. However, under all other conditions, it remains highly conservative. In contrast, the Wald-Obs statistic shows a clear advantage under moderate quality items, yielding good empirical Type I error rates under most conditions.

**Table 6:** The empirical Type I error rates for Wald-XPD, Wald-Obs, and LR when *K* = 5 and items were of moderate quality ( α=0.05 ).

		Uniform			Non-uniform		
Structure	*N*	W-XPD	W-Obs	LR	W-XPD	W-Obs	LR
Linear	200	0.012	**0.046**	0	0.022	**0.057**	0.002
	500	0.002	0.076	0.002	0.002	**0.068**	0.002
	1,000	0	**0.059**	0.002	0	**0.042**	0.004
Inverted pyramid	200	0.008	**0.064**	0.012	0.014	**0.052**	0.01
	500	0	0.025	0.008	0	**0.038**	0.01
	1,000	0	**0.036**	0.002	0	**0.038**	0.006
Pyramid	200	0.004	0.217	0.218	0.012	0.079	0.06
	500	0.002	**0.065**	0.028	0	**0.034**	0.014
	1,000	0	**0.047**	**0.048**	0	0.021	0.016
Diamond	200	0.016	0.079	**0.036**	0.008	0.079	0.02
	500	0	**0.038**	0.016	0	**0.05**	0
	1,000	0	**0.059**	0.008	0	**0.047**	0.002

The bold font represents an acceptable empirical Type I error rates at a significance level of 0.05.

Table 7 presents the average empirical Type I error rates for low-quality items when *K =* 5. It can be seen that under low-quality items, especially with a uniform population distribution, the Wald-XPD statistic shows improvement in some conditions. For instance, when *N* = 200, the Wald-XPD statistic controls the empirical Type I error rates close to the nominal level of 0.05 when testing the pyramid and inverted pyramid hierarchies. Under non-uniform distribution, the Wald-XPD statistic yields empirical Type I error rates close to 0.05 for linear and diamond structures at *N* = 200, and performs well for pyramid and diamond structures at *N* = 500. In contrast, both the Wald-Obs and LR statistics consistently exhibit very conservative Type I error rates under low-quality items.

**Table 7:** The empirical Type I error rates for Wald-XPD, Wald-Obs, and LR when *K* = 5 and items were of low quality ( α=0.05 ).

		Uniform			Non-uniform		
Structure	*N*	W-XPD	W-Obs	LR	W-XPD	W-Obs	LR
Linear	200	**0.032**	0.423	0.492	**0.044**	0.515	0.556
	500	0.018	0.134	0.182	0.024	0.185	0.258
	1,000	0	0.095	0.108	0	0.073	0.122
Inverted pyramid	200	**0.038**	0.512	0.664	0.016	0.389	0.518
	500	0.07	0.21	0.334	0.014	0.162	0.296
	1,000	0.002	**0.059**	0.102	0	0.094	0.15
Pyramid	200	**0.038**	**0.58**	0.72	0.014	0.477	0.614
	500	0.22	0.331	0.596	**0.064**	0.274	0.444
	1,000	0.134	0.087	0.284	0.022	0.095	0.208
Diamond	200	0.078	0.595	0.746	**0.034**	0.514	0.582
	500	0.188	0.278	0.468	**0.05**	0.181	0.348
	1,000	**0.032**	**0.085**	0.162	0.008	0.087	0.142

The bold font represents an acceptable empirical Type I error rates at a significance level of 0.05.

In general, regarding the empirical Type I error rate, all three statistics are affected by item quality. When item quality was high, the LR test appeared to be overly conservative compared to Wald-XPD and Wald-Obs. When item quality was low, the LR test had an inflated Type I error rate. In contrast, Wald_XPD seems like a feasible alternative in these scenarios. For moderate-quality items, when K = 3, LR performs better in some conditions, while Wald-XPD is overly conservative and Wald-Obs shows inflated Type I error rates. When K = 5, Wald-Obs produces better empirical Type I error rates in most conditions and significantly outperforms the LR statistic.

### 3.2 Simulation study 2

#### 3.2.1 Design

In Simulation Study 2, the factorial design was the same as in Study 1. However, the generating and fitted models were different. For both statistical test methods, the GDINA model with identity link was used to generate the data. For the Wald test, the fitted model was consistent with the data generation model, whereas the HDCM with identity links was used to fit the data in the LR test.

Study 2 evaluated the power of these statistical testing procedures (Cohen, 1992). Statistical power refers to the percentage of correctly rejecting the null hypothesis when it is not correct in *n* replications. When an attribute hierarchy exists, if the Wald statistic follows an asymptotic chi-square distribution, the observed Type-I error rate should conform to a pre-set theoretical Type-I error rate, such as 0.05. If there is no attribute hierarchy, the larger the proportion of the Wald statistic that correctly rejects the null hypothesis, the stronger the confidence that it can correctly test for the absence of an attribute hierarchy. As in de la Torre and Lee (2013) and Ma and de la Torre (2019), a test power of at least 0.80 is sufficient and greater than or equal to 0.90 is excellent.

#### 3.2.2 Results

Table 8 presents the statistical power of each test method when *K* = 3 across various conditions. It can be seen that under the uniform population distribution, all three methods show strong performance, with statistical power equal to 1 or very close to 1 in the case of high and medium item quality. However, for low-quality items with *N* = 200, the power of the Wald-Obs and Wald-XPD statistics was less than 0.99 when testing the pyramid and inverted pyramid attribute hierarchies. Under non-uniform distribution, all methods perform well with high quality items, with power higher than 0.80. At moderate and low item quality, the statistical power of Wald-XPD and Wald-Obs remains stable above 0.80, while Wald-XPD shows more variability, especially at low quality items, with power less than 0.7 at a sample size of 200. Overall, LR and Wald-Obs are more reliable, and Wald-XPD is more sensitive to lower item quality, especially at sample sizes of 200.

**Table 8:** The statistical power for Wald-XPD, Wald-Obs, and LR when *K* = 3.

			Uniform			Non-uniform		
IQ	Structure	*N*	W-XPD	W-Obs	LR	W-XPD	W-Obs	LR
High	Linear	200	1	1	1	0.97	1	1
		500	1	1	1	1	1	1
		1,000	1	1	1	1	1	1
	Inverted pyramid	200	1	1	1	0.996	0.996	1
		500	1	1	1	1	1	1
		1,000	1	1	1	1	1	1
	Pyramid	200	1	1	1	0.964	1	1
		500	1	1	1	1	1	1
		1,000	1	1	1	1	1	1
Moderate	Linear	200	1	0.998	1	0.968	0.994	1
		500	1	1	1	1	1	1
		1,000	1	1	1	1	1	1
	Inverted pyramid	200	1	1	1	0.914	0.99	1
		500	1	1	1	1	1	1
		1,000	1	1	1	1	1	1
	Pyramid	200	1	1	1	0.766	0.994	1
		500	1	1	1	1	1	1
		1,000	1	1	1	1	1	1
Low	Linear	200	0.996	0.998	1	0.578	0.97	0.99
		500	1	1	1	0.998	0.983	1
		1,000	1	1	1	1	0.998	1
	Inverted pyramid	200	0.964	0.982	0.996	0.458	0.925	0.946
		500	1	0.992	1	0.956	0.953	0.998
		1,000	1	1	1	1	0.992	1
	Pyramid	200	0.97	0.985	0.996	0.658	0.963	0.986
		500	1	1	1	1	0.981	1
		1,000	1	1	1	1	0.998	1

IQ represents item quality; structure represents attribute hierarchy type; *N* is the sample size. LR is the likelihood ratio test; W-Obs is the Wald statistic calculated using the observed information matrix; W-XPD is the Wald statistic calculated using the empirical cross-product matrix; uniform is the uniform population distribution; non-uniform is the non-uniform population distribution. Linear is a linear hierarchy; inverted pyramid is an inverted pyramid hierarchy; pyramid is a pyramid hierarchy.

Table 9 shows the statistical power of the three tests under various conditions when *K* = 5. Under the uniform distribution condition, the statistical power of the three methods is very high (close to 1) for both high- and medium-quality items, whereas the statistical power of Wald-XPD and Wald-Obs appears to be significantly reduced for low-quality items, especially when the Wald-XPD statistic tests the pyramid structure down to 0.408 at *N* = 200, which may be due to the large number of attributes and small sample size leads to bias in parameter estimation, which affects the covariance estimation of structural parameters as well as the performance of the Wald test. Similarly, the statistical power of Wald-XPD is very small at a sample size of 200 under non-uniform conditions, which may indicate that the test is prone to failure under small samples. In contrast, the LR and Wald-Obs statistics show more stable performance and excellent statistical power. In general, the empirical power of the LR statistic is consistently better than the other methods under all simulation conditions, and Wald-XPD and Wald-Obs are also an alternative in large samples (N > 200). Regarding sample size, Wald-Obs and LR perform more consistently across samples, while Wald-XPD performs poorly under small sample conditions. For attribute distributions, all three statistics have better statistical power under uniform distribution conditions than non-uniform distribution. For item quality, with a few exceptions, all three statistics show good power for high and medium quality items. For low-quality items, the empirical power of the Wald-XPD and Wald-Obs statistics decreases significantly in most cases.

**Table 9:** The statistical power for Wald-XPD, Wald-Obs, and LR when *K* = 5.

			Uniform			Non-uniform		
IQ	Structure	*N*	W-XPD	W-Obs	LR	W-XPD	W-Obs	LR
High	Linear	200	1	0.998	1	1	0.994	1
		500	1	1	1	1	1	1
		1,000	1	1	1	1	1	1
	Inverted pyramid	200	1	1	1	0.802	0.964	1
		500	1	1	1	1	0.998	1
		1,000	1	1	1	1	1	1
	Pyramid	200	0.998	0.996	1	0.518	0.967	1
		500	1	1	1	1	1	1
		1,000	1	1	1	1	1	1
	Diamond	200	1	0.998	1	0.786	0.967	1
		500	1	1	1	1	1	1
		1,000	1	1	1	1	1	1
Moderate	Linear	200	1	0.993	1	0.886	0.953	1
		500	1	1	1	1	1	1
		1,000	1	1	1	1	1	1
	Inverted pyramid	200	0.998	0.994	1	0.17	0.88	1
		500	1	1	1	1	0.998	1
		1,000	1	1	1	1	1	1
	Pyramid	200	0.956	0.96	1	0.188	0.858	1
		500	1	1	1	1	0.994	1
		1,000	1	1	1	1	1	1
	Diamond	200	1	0.98	1	0.684	0.869	1
		500	1	1	1	1	0.996	1
		1,000	1	1	1	1	1	1
Low	Linear	200	0.984	0.957	1	0.498	0.929	1
		500	1	0.985	1	1	0.974	1
		1,000	1	1	1	1	0.996	1
	Inverted pyramid	200	0.766	0.902	1	0.166	0.833	0.962
		500	1	0.966	1	0.944	0.876	1
		1,000	1	1	1	1	0.984	1
	Pyramid	200	0.408	0.859	0.978	0.078	0.747	0.906
		500	0.968	0.846	1	0.812	0.819	0.986
		1,000	1	0.952	1	0.996	0.951	1
	Diamond	200	0.816	0.92	1	0.276	0.859	0.996
		500	1	0.964	1	0.982	0.925	1
		1,000	1	0.996	1	1	0.986	1

To evaluate the computational efficiency of different attribute hierarchy testing methods, Tables S1, S2 in the Appendix summarize the average runtimes of the parameter estimation based on the EM algorithm, as well as the average runtime of the three testing methods (including covariance matrix estimation, test statistic computation, and hypothesis testing). From Tables S1 and S2, it can be observed that the time-consumption of both the parameter estimation and attribute hierarchy testing procedures increases with the sample size, leading to a decrease in computational efficiency. Comparing the computational efficiency of different attribute hierarchy testing methods, the Wald-XPD method has the shortest runtime and the highest computational efficiency, followed by the LR statistic. In contrast, the Wald-Obs method has the lowest testing efficiency, with a computation time of up to 36 s. This result is expected, according to Liu et al. (2019b), the calculation of the information matrix in CDM requires traversing all observable response patterns of the test-takers. Therefore, as the sample size increases, the number of observable response patterns also increases, leading to greater computational complexity, longer computation times, and lower efficiency. Moreover, the computation of the Obs matrix includes the XPD matrix, making the Wald-Obs method more time-consuming and less efficient than the Wald-XPD method. It is worth noting that the results of the hypothesis testing can be used to guide model selection, which can further estimate examinees’ attribute mastery patterns, and facilitate examinee classification. The results of classification accuracy under different attribute hierarchy testing methods are presented in Tables S3 and S4 in the Appendix. Table S3 shows that examinees’ classification accuracy is affected by item quality and sample size: higher item quality and larger sample size result in higher classification accuracy. Comparison of the different attribute hierarchy tests reveals that all three methods show consistent accuracy under high quality items, under medium quality items, Wald-XPD and LR perform similarly and both slightly outperform Wald-Obs, and under low quality items, Wald-XPD outperforms the Wald-Obs and LR methods in terms of classification accuracy. Similar results are shown in Table S4, and the classification accuracy of each method shows a significant decrease as the number of attributes increases.

## 4 Real data examples

### 4.1 Data and analysis

**Figure 2:**
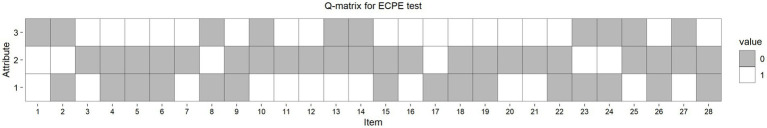
*Q*-matrix for the ECPE.

This section provides a practical illustration of how to validate the attribute hierarchy of the English Certificate of Proficiency Examination (ECPE; Templin and Hoffman, 2013) dataset using the Wald-XPD and Wald-Obs statistics. The dataset is available directly in the R package CDM (Robitzsch et al., 2022). The ECPE dataset contains binary responses from 2,922 examinees to 28 items on the grammar section of the ECPE. These items are designed to measure three attributes: the application of (a) morphosyntactic rules ( $\alpha_{1}$ ), (b) cohesive rules ( $\alpha_{2}$ ), and (c) lexical rules ( $\alpha_{3}$ ; Buck and Tatsuoka, 1998). Figure 2 shows the *Q* matrix for ECPE from Templin and Bradshaw’s (2014) study.

The dataset has now been investigated as a common example in many DCM applications, and many researchers (e.g., Templin and Hoffman, 2013; Templin and Bradshaw, 2014; Liu et al., 2022; Wang and Lu, 2021) have demonstrated the existence of a linear hierarchical structure in the three attributes that ECPE examines. Specifically, the mastery of lexical rules ( $\alpha_{3}$ ) is a prerequisite for the mastery of cohesive rules ( $\alpha_{2}$ ), and the mastery of cohesive rules is a prerequisite ( $\alpha_{2}$ ) for the mastery of morphosyntactic rules ( $\alpha_{1}$ ). The present study verified whether the proposed methods were effective in detecting a linear hierarchical structure ( $\alpha_{3}\to \alpha_{2}\to \alpha_{1}$ ) in the ECPE.

First, the G-DINA model with identity link function was used to fit the ECPE dataset and the MMLE-EM algorithm was used to estimate all structural and item parameter estimates. Then, the covariance matrices of all structural parameters were estimated using XPD and Obs estimators. The structural parameter vectors to be tested were determined based on a linear attribute hierarchy defined *a priori*. Further, the Wald statistic was computed and a hypothesis test for attribute hierarchies was performed. The null hypothesis here is that all structural parameter vector estimates to be tested are not significantly different from zero. In the ECPE data, attribute mastery patterns $\alpha_{2}={\left(1,0,0\right)}^{\prime }$ , $\alpha_{3}={\left(0,1,0\right)}^{\prime }$ , $\alpha_{5}={\left(1,1,0\right)}^{\prime }$ , and $\alpha_{6}={\left(1,0,1\right)}^{\prime }$ are impermissible if the three attributes followed a linear hierarchical relationship, that is, structural parameters $\pi_{2}$ , $\pi_{3}$ , $\pi_{5}$ and $\pi_{6}$ of the saturated models that should not be significantly larger than zero. Therefore, for the Wald test, the set of structural parameters to be tested is $\hat{\pi }=\left(\pi_{2},\pi_{3},\pi_{5},\pi_{6}\right)$ . At a pre-specified level of significance, if the Wald statistic fails to reject the null hypothesis, it indicates that there is a linear hierarchy of attributes in the data.

### 4.2 Results

In order to investigate the consistency of the Wald statistic with the LR statistic for testing attribute hierarchies, an HDCM with a linear hierarchy was also used to fit the ECPE data. The example of this LR test is based on previous work by Templin and Bradshaw et al. (2014), who specified HDCM for the item parameters and structural parameters in their study and verified the presence of a linear attribute hierarchy in the data by performing the LR test for saturated model and HDCM.

Table 10 provides the values of the Wald-XPD and Wald-Obs statistics and LR statistics and their corresponding *p*-values. From Table 10, it can be observed that the Wald-XPD, Wald-Obs, and LR statistics perform very similarly with their p-values of 0.017, 0.020, and 0.020, respectively. From the simulation study, we know that these *p*-values are very conservative, so if we select a significance level of 0.05, we would be very confident in rejecting the null hypothesis and concluding that there is no linear attribute hierarchy in the data, whereas by selecting a significance level of 0.01, we would fail to reject the null hypothesis and conclude that there is a hierarchical structure of attributes in these data. This result is similar to the results of a previous study by Templin and Bradshaw et al. (2014). In the simulation study, the performance of each method at *K* = 3 is affected by the quality of the items. For example, at high quality, both the Wald-XPD and LR statistics exhibit very conservative empirical Type I error rates but have excellent power. Although it is difficult to know the quality of the items in the ECPE data, by combining the performance of the Wald-XPD and Wald-Obs statistic in the simulation study, it is further possible to demonstrate the existence of a linear hierarchical structure in the ECPE data. For example, in the case of high quality items, both the Wald-XPD and LR statistics exhibit very conservative empirical Type I error rates, but have excellent power, which also leads to the conclusion that there is a linear attribute hierarchy.

**Table 10:** Results obtained for the Wald statistics and LR.

Method	Value	df	*p*
$W_{XPD}$	12.032	4	0.017
$W_{Obs}$	11.678	4	0.020
*LR*	25.509	13	0.020

df denotes the degrees of freedom, and *p* is the significance level.

## 5 Conclusions and discussion

Validating attribute hierarchies has important theoretical and practical implications for test development and diagnostic evaluation. Most of the early attribute hierarchies were obtained through theoretical analysis by domain experts, which are somewhat subjective. Previous studies have found that attribute hierarchy specification directly affects the accuracy of item-level and test-level model-data fitting, item parameter estimation, and examinee classification (Liu et al., 2017; Liu, 2018; Tu et al., 2019), and this negative impact cannot be compensated for by carefully setting up the test items or Q matrix. In view of this, it is necessary to provide evidence that the attribute hierarchy hypothesized by the researcher is supported by theoretical constructs and statistical evidence (Bradshaw et al., 2014). For the validation of the attribute hierarchy, researchers usually use the LR test based on the HDCM model. Due to the lack of regularity, the asymptotic distribution of the LR test becomes non-standardized, and Ma and Xu (2021) found that when the number of items is large or the item parameters are close to the boundaries, the non-standard limiting distributions converge very slowly, leading to possible failure of the hypothesis test. The z-statistic for attribute hierarchy test proposed by Liu et al. (2022) is susceptible to the accuracy of standard error estimation in its computation, and this method requires a cumbersome process of testing the structural parameters one by one. Therefore, this study proposes the Wald statistic to statistically validate the a priori defined attribute hierarchy. A simulation study and empirical data are used to assess the empirical performance of the Wald statistic and LR statistic in testing attribute hierarchies. Practically, this study aims to provide a set of tools that can be used with the CDM to provide researchers with a new way of thinking and an alternative approach when conducting attribute hierarchy tests for the CDM.

Simulation studies have shown that the LR test is overly conservative in terms of empirical Type I error rates when item quality is high, while the LR test produces more inflated Type I error rates when item quality is low. In contrast, Wald_XPD seems to be an alternative in these cases. In terms of statistical power, when the sample size is greater than 200, the statistical power of all three methods is excellent and robust in most cases. In terms of computational efficiency, the Wald-XPD method demonstrates a significant computational efficiency advantage. In contrast, the LR test is slightly less computationally efficient than the Wald-XPD method due to the complex parameter iteration process involved. In addition, a preliminary investigation of the computational efficiency of the Bootstrap-based Wald test method was conducted under the condition that each of the 500 independently repeated experiments contained 50 resamples, and it was found that the average time consumed for the complete test was 6.293, 15.116, and 44.999 s for the sample sizes of 200, 500, and 1,000, respectively. It can be seen that the larger the sample size, the higher the computational cost of the resampling method and the longer the processing time. These results confirm the warnings in the literature regarding the computational intensity of resampling methods (Ma and Xu, 2021; Liu et al., 2022), particularly when handling large-scale data, where the time cost may become prohibitive. In addition, this study referred to the experimental design of Ma and Xu (2021), which considered the length of the 30-item test and could represent the long test situation. It was found that in this case, the two Wald statistics proposed in this study have some advantages in some cases compared to LR, and these results further verify the findings of Ma and Xu (2021) regarding the LR test. In addition, the simulation study found that, the LR test has a very small statistical power at *K* = 5 with small samples, and we know that the smaller the power, the larger the p-value, which implies that the incorrect null hypotheses that we would not reject. This may be due to the fact that in statistical power analysis, we assume that all latent attribute profiles are present in the data generation process and that the distribution of true attribute mastery patterns is nonuniform distribution. When the number of attributes is large, the number of attribute mastery patterns is also large, and when the sample size is small, some attribute mastery patterns may be null, and the parameter estimation may be severely biased, and the bias in parameter estimation will be further transmitted to the LR test process, leading to test failure. In summary, we recommend using Wald-XPD for attribute stratification testing in long tests.

Although the manuscript has yielded some promising findings, there are still some valuable issues worth investigating further. First, this study does not explore the performance of the new method with a large number of attributes. Due to the increase in the number of attributes, the structural parameters that need to be estimated will grow exponentially, in which case there is a challenge of high-dimensional estimation, and the stability and accuracy of the existing attribute hierarchy testing methods deserve to be further explored. Second, for the variables manipulated, it was found that there is little difference in the statistical results obtained by the various methods for different attribute structures. Many other factors that may affect attribute hierarchy validation were not addressed in this study. For example, the authors found factors such as test length, fitted model, and correct/incorrect specification of the Q matrix to be valuable in examining how these factors affect the statistical properties of the Wald statistic used to validate the attribute hierarchy. Additionally, a follow-up question of interest is how to estimate the attribute mastery patterns of examinees and classify examinees after validating the attribute hierarchy, researchers have proposed an attribute hierarchy-based approach to CDM parameter estimation (Akbay and de la Torre, 2020; Tu et al., 2019), and the effect of the specification of the attribute hierarchy on these methods remains to be further investigated.

## Data Availability

Publicly available datasets were analyzed in this study. This data can be found at: https://CRAN.R-project.org/package=CDM.
