# Supplementary material for: Validating attribute hierarchies in cognitive diagnosis models
Source: Front Psychol. 2025 Apr 28;16:1562807. doi: 10.3389/fpsyg.2025.1562807 (PMC12067792; doi:10.3389/fpsyg.2025.1562807)
Supplement: Supplementary file 1 [file Table_1.docx]

# Appendix material

Tables S1 and S2 provide the average running time of the three attribute hierarchy testing methods under different experimental conditions for *K* = 3 and *K* = 5, respectively, as well as the time (in seconds) spent on parameter estimation using the EM algorithm.

Table S 1 Summary of average running times (in seconds) for parameter estimation, W-XPD, W-Obs and LR tests when *K* = 3

|  |  |  | ***Uniform*** | | | | ***Non-uniform*** | | | |
| --- | --- | --- | --- | --- | --- | --- | --- | --- | --- | --- |
| ***IQ*** | ***Structure*** | ***N*** | ***EM*** | ***W-XPD*** | ***W-Obs*** | ***LR*** | ***EM*** | ***W-XPD*** | ***W-Obs*** | ***LR*** |
| High | Linear | 200 | 0.061 | 0.016 | 0.55 | 0.033 | 0.064 | 0.016 | 0.519 | 0.031 |
|  |  | 500 | 0.11 | 0.041 | 1.355 | 0.053 | 0.118 | 0.041 | 1.3 | 0.053 |
|  |  | 1000 | 0.439 | 0.178 | 6.098 | 0.204 | 0.425 | 0.171 | 5.753 | 0.191 |
|  | Inverted pyramid | 200 | 0.041 | 0.012 | 0.305 | 0.024 | 0.294 | 0.069 | 1.973 | 0.175 |
|  |  | 500 | 0.046 | 0.021 | 0.531 | 0.028 | 0.332 | 0.11 | 3.306 | 0.194 |
|  |  | 1000 | 0.06 | 0.038 | 0.841 | 0.041 | 0.225 | 0.1 | 3.643 | 0.129 |
|  | Pyramid | 200 | 0.27 | 0.046 | 1.33 | 0.193 | 0.186 | 0.047 | 1.366 | 0.11 |
|  |  | 500 | 0.294 | 0.135 | 4.766 | 0.17 | 0.209 | 0.05 | 1.37 | 0.121 |
|  |  | 1000 | 0.324 | 0.121 | 3.861 | 0.188 | 0.27 | 0.144 | 5.04 | 0.173 |
| Moderate | Linear | 200 | 0.111 | 0.019 | 0.615 | 0.053 | 0.1 | 0.018 | 0.583 | 0.049 |
|  |  | 500 | 0.249 | 0.05 | 1.587 | 0.086 | 0.233 | 0.047 | 1.454 | 0.077 |
|  |  | 1000 | 1.006 | 0.2 | 6.74 | 0.271 | 0.926 | 0.192 | 6.398 | 0.262 |
|  | Inverted pyramid | 200 | 0.429 | 0.071 | 2.043 | 0.291 | 0.483 | 0.069 | 2.014 | 0.327 |
|  |  | 500 | 0.456 | 0.11 | 3.414 | 0.236 | 0.522 | 0.105 | 3.166 | 0.315 |
|  |  | 1000 | 0.308 | 0.096 | 3.513 | 0.143 | 0.326 | 0.089 | 3.206 | 0.159 |
|  | Pyramid | 200 | 0.897 | 0.056 | 1.66 | 0.769 | 0.619 | 0.079 | 2.657 | 0.294 |
|  |  | 500 | 1.417 | 0.097 | 3.185 | 0.893 | 0.823 | 0.072 | 2.432 | 0.497 |
|  |  | 1000 | 1.602 | 0.1 | 3.111 | 0.943 | 0.892 | 0.056 | 1.595 | 0.625 |
| Low | Linear | 200 | 0.274 | 0.019 | 0.626 | 0.183 | 0.263 | 0.019 | 0.597 | 0.199 |
|  |  | 500 | 0.827 | 0.069 | 2.152 | 0.366 | 0.808 | 0.065 | 1.976 | 0.311 |
|  |  | 1000 | 1.927 | 0.182 | 6.318 | 0.519 | 2.122 | 0.18 | 6.115 | 0.528 |
|  | Inverted pyramid | 200 | 1.158 | 0.072 | 2.032 | 0.935 | 1.113 | 0.07 | 1.995 | 0.762 |
|  |  | 500 | 0.989 | 0.076 | 2.527 | 0.676 | 1.106 | 0.081 | 2.526 | 0.743 |
|  |  | 1000 | 0.451 | 0.062 | 1.919 | 0.214 | 0.606 | 0.059 | 1.879 | 0.379 |
|  | Pyramid | 200 | 0.448 | 0.125 | 4.364 | 0.223 | 0.324 | 0.049 | 1.363 | 0.218 |
|  |  | 500 | 0.521 | 0.124 | 3.912 | 0.269 | 0.37 | 0.121 | 3.694 | 0.196 |
|  |  | 1000 | 0.593 | 0.123 | 3.712 | 0.353 | 0.419 | 0.138 | 4.816 | 0.208 |
| ***Notes***. *EM* stands for the average parameter estimation time of the EM algorithm, *W-XPD* stands for the average running time of the Wald-XPD test procedure, *W-Obs* stands for the average running time of the Wald-Obs test procedure, and LR stands for the average running time of the likelihood ratio test procedure | | | | | | | | | | |

Table S 2 Summary of average running times (in seconds) for parameter estimation, W-XPD, W-Obs and LR tests when *K* = 5

|  |  |  | ***Uniform*** | | | | ***Non-uniform*** | | | |
| --- | --- | --- | --- | --- | --- | --- | --- | --- | --- | --- |
| ***IQ*** | ***Structure*** | ***N*** | ***EM*** | ***W-XPD*** | ***W-Obs*** | ***LR*** | ***EM*** | ***W-XPD*** | ***W-Obs*** | ***LR*** |
| High | Linear | 200 | 0.397 | 0.073 | 6.945 | 0.125 | 0.313 | 0.068 | 5.328 | 0.125 |
|  |  | 500 | 0.518 | 0.199 | 19.509 | 0.141 | 0.498 | 0.145 | 13.797 | 0.144 |
|  |  | 1000 | 0.776 | 0.34 | 34.114 | 0.182 | 0.767 | 0.288 | 28.022 | 0.183 |
|  | Inverted pyramid | 200 | 0.325 | 0.066 | 6.267 | 0.128 | 0.301 | 0.062 | 4.884 | 0.137 |
|  |  | 500 | 0.46 | 0.186 | 18.29 | 0.15 | 0.423 | 0.143 | 13.323 | 0.156 |
|  |  | 1000 | 0.725 | 0.343 | 34.528 | 0.195 | 0.617 | 0.286 | 26.344 | 0.205 |
|  | Pyramid | 200 | 0.39 | 0.073 | 7.195 | 0.164 | 0.336 | 0.07 | 5.699 | 0.182 |
|  |  | 500 | 0.522 | 0.197 | 18.609 | 0.173 | 0.468 | 0.142 | 13.009 | 0.221 |
|  |  | 1000 | 0.764 | 0.336 | 33.296 | 0.218 | 0.662 | 0.277 | 25.511 | 0.288 |
|  | Diamond | 200 | 0.336 | 0.062 | 5.147 | 0.124 | 0.282 | 0.051 | 3.444 | 0.129 |
|  |  | 500 | 0.572 | 0.202 | 19.619 | 0.156 | 0.477 | 0.144 | 13.667 | 0.191 |
|  |  | 1000 | 0.771 | 0.349 | 36.003 | 0.195 | 0.752 | 0.287 | 28.336 | 0.23 |
| Moderate | Linear | 200 | 0.799 | 0.07 | 7.296 | 0.243 | 0.73 | 0.064 | 5.641 | 0.23 |
|  |  | 500 | 1.278 | 0.201 | 19.986 | 0.209 | 1.165 | 0.146 | 13.949 | 0.182 |
|  |  | 1000 | 2.053 | 0.307 | 32.568 | 0.244 | 2.176 | 0.28 | 27.186 | 0.226 |
|  | Inverted pyramid | 200 | 0.802 | 0.068 | 6.922 | 0.408 | 0.727 | 0.064 | 5.327 | 0.413 |
|  |  | 500 | 1.168 | 0.197 | 19.923 | 0.269 | 1.232 | 0.148 | 13.982 | 0.468 |
|  |  | 1000 | 1.794 | 0.32 | 33.097 | 0.3 | 2.066 | 0.282 | 26.537 | 0.587 |
|  | Pyramid | 200 | 1.043 | 0.076 | 7.721 | 0.581 | 0.23 | 0.019 | 0.882 | 0.147 |
|  |  | 500 | 1.311 | 0.194 | 19.94 | 0.359 | 0.373 | 0.034 | 1.588 | 0.211 |
|  |  | 1000 | 1.655 | 0.308 | 30.263 | 0.349 | 0.73 | 0.065 | 3.205 | 0.317 |
|  | Diamond | 200 | 0.86 | 0.064 | 6.184 | 0.381 | 0.716 | 0.057 | 4.577 | 0.369 |
|  |  | 500 | 1.327 | 0.194 | 19.913 | 0.268 | 1.329 | 0.143 | 13.854 | 0.502 |
|  |  | 1000 | 1.837 | 0.336 | 34.023 | 0.284 | 2.125 | 0.28 | 27.282 | 0.624 |
| Low | Linear | 200 | 1.628 | 0.069 | 7.229 | 0.839 | 1.452 | 0.065 | 5.597 | 0.82 |
|  |  | 500 | 3.168 | 0.192 | 18.733 | 0.711 | 3.075 | 0.141 | 13.735 | 0.704 |
|  |  | 1000 | 4.403 | 0.28 | 27.777 | 0.458 | 4.981 | 0.265 | 23.584 | 0.516 |
|  | Inverted pyramid | 200 | 1.695 | 0.063 | 6.871 | 1.375 | 1.526 | 0.063 | 5.266 | 1.15 |
|  |  | 500 | 3.834 | 0.19 | 18.905 | 1.898 | 3.399 | 0.137 | 13.788 | 1.94 |
|  |  | 1000 | 4.576 | 0.243 | 23.775 | 0.963 | 5.116 | 0.232 | 20.182 | 2.098 |
|  | Pyramid | 200 | 1.756 | 0.07 | 7.548 | 1.479 | 1.578 | 0.07 | 6.311 | 1.241 |
|  |  | 500 | 4.642 | 0.184 | 19.491 | 2.721 | 3.668 | 0.142 | 14.526 | 2.252 |
|  |  | 1000 | 5.639 | 0.207 | 19.024 | 1.991 | 5.239 | 0.205 | 17.39 | 2.851 |
|  | Diamond | 200 | 1.624 | 0.062 | 6.244 | 1.163 | 1.657 | 0.06 | 4.893 | 1.091 |
|  |  | 500 | 4.464 | 0.19 | 19.274 | 1.746 | 4.137 | 0.147 | 14.325 | 1.759 |
|  |  | 1000 | 5.262 | 0.252 | 25.393 | 0.975 | 5.9 | 0.233 | 20.813 | 2.187 |

Tables S3 and S4 present the classification accuracy results of examinees obtained by the three attribute hierarchy testing methods under different experimental conditions for *K* = 3 and *K* = 5, respectively.

Table S 3 Classification accuracy results for the three attribute hierarchy test methods when *K* = 3

|  |  |  | ***Uniform*** | | | ***Non-uniform*** | | |
| --- | --- | --- | --- | --- | --- | --- | --- | --- |
| ***IQ*** | ***Structure*** | ***N*** | ***W-XPD*** | ***W-Obs*** | ***LR*** | ***W-XPD*** | ***W-Obs*** | ***LR*** |
| High | Linear | 200 | 0.987 | 0.987 | 0.987 | 0.987 | 0.987 | 0.987 |
|  |  | 500 | 0.989 | 0.988 | 0.989 | 0.988 | 0.988 | 0.988 |
|  |  | 1000 | 0.989 | 0.989 | 0.989 | 0.988 | 0.988 | 0.988 |
|  | Inverted pyramid | 200 | 0.979 | 0.979 | 0.979 | 0.981 | 0.981 | 0.981 |
|  |  | 500 | 0.981 | 0.981 | 0.981 | 0.983 | 0.983 | 0.983 |
|  |  | 1000 | 0.982 | 0.982 | 0.982 | 0.985 | 0.985 | 0.985 |
|  | Pyramid | 200 | 0.979 | 0.979 | 0.979 | 0.98 | 0.98 | 0.98 |
|  |  | 500 | 0.981 | 0.981 | 0.981 | 0.983 | 0.983 | 0.983 |
|  |  | 1000 | 0.982 | 0.982 | 0.982 | 0.984 | 0.984 | 0.984 |
| Moderate | Linear | 200 | 0.914 | 0.898 | 0.912 | 0.911 | 0.898 | 0.909 |
|  |  | 500 | 0.924 | 0.915 | 0.922 | 0.92 | 0.911 | 0.918 |
|  |  | 1000 | 0.925 | 0.919 | 0.924 | 0.923 | 0.917 | 0.921 |
|  | Inverted pyramid | 200 | 0.874 | 0.872 | 0.874 | 0.886 | 0.88 | 0.885 |
|  |  | 500 | 0.89 | 0.889 | 0.889 | 0.9 | 0.898 | 0.899 |
|  |  | 1000 | 0.894 | 0.894 | 0.894 | 0.906 | 0.904 | 0.905 |
|  | Pyramid | 200 | 0.873 | 0.87 | 0.872 | 0.885 | 0.879 | 0.884 |
|  |  | 500 | 0.889 | 0.887 | 0.889 | 0.899 | 0.898 | 0.899 |
|  |  | 1000 | 0.893 | 0.892 | 0.893 | 0.905 | 0.904 | 0.905 |
| Low | Linear | 200 | 0.689 | 0.608 | 0.628 | 0.681 | 0.598 | 0.621 |
|  |  | 500 | 0.74 | 0.702 | 0.711 | 0.734 | 0.7 | 0.707 |
|  |  | 1000 | 0.764 | 0.743 | 0.746 | 0.758 | 0.738 | 0.74 |
|  | Inverted pyramid | 200 | 0.601 | 0.55 | 0.568 | 0.648 | 0.586 | 0.605 |
|  |  | 500 | 0.663 | 0.643 | 0.652 | 0.704 | 0.676 | 0.689 |
|  |  | 1000 | 0.692 | 0.682 | 0.687 | 0.733 | 0.721 | 0.727 |
|  | Pyramid | 200 | 0.594 | 0.548 | 0.564 | 0.637 | 0.569 | 0.59 |
|  |  | 500 | 0.663 | 0.643 | 0.649 | 0.69 | 0.665 | 0.677 |
|  |  | 1000 | 0.692 | 0.683 | 0.687 | 0.718 | 0.707 | 0.712 |

Table S 4 Classification accuracy results for the three attribute hierarchy test methods when *K* = 5

|  |  |  | ***Uniform*** | | | ***Non-uniform*** | | |
| --- | --- | --- | --- | --- | --- | --- | --- | --- |
| ***IQ*** | ***Structure*** | ***N*** | ***W-XPD*** | ***W-Obs*** | ***LR*** | ***W-XPD*** | ***W-Obs*** | ***LR*** |
| High | Linear | 200 | 0.957 | 0.959 | 0.959 | 0.958 | 0.959 | 0.96 |
|  |  | 500 | 0.962 | 0.962 | 0.962 | 0.961 | 0.961 | 0.962 |
|  |  | 1000 | 0.964 | 0.964 | 0.964 | 0.963 | 0.962 | 0.963 |
|  | Inverted pyramid | 200 | 0.932 | 0.932 | 0.933 | 0.941 | 0.941 | 0.941 |
|  |  | 500 | 0.939 | 0.939 | 0.939 | 0.945 | 0.945 | 0.945 |
|  |  | 1000 | 0.942 | 0.942 | 0.942 | 0.947 | 0.947 | 0.947 |
|  | Pyramid | 200 | 0.914 | 0.913 | 0.914 | 0.935 | 0.936 | 0.936 |
|  |  | 500 | 0.922 | 0.921 | 0.922 | 0.942 | 0.942 | 0.942 |
|  |  | 1000 | 0.925 | 0.925 | 0.925 | 0.944 | 0.944 | 0.944 |
|  | Diamond | 200 | 0.934 | 0.934 | 0.935 | 0.944 | 0.945 | 0.945 |
|  |  | 500 | 0.939 | 0.94 | 0.94 | 0.949 | 0.949 | 0.949 |
|  |  | 1000 | 0.942 | 0.942 | 0.942 | 0.95 | 0.95 | 0.95 |
| Moderate | Linear | 200 | 0.819 | 0.815 | 0.82 | 0.827 | 0.824 | 0.827 |
|  |  | 500 | 0.839 | 0.837 | 0.838 | 0.842 | 0.839 | 0.842 |
|  |  | 1000 | 0.843 | 0.843 | 0.843 | 0.848 | 0.847 | 0.848 |
|  | Inverted pyramid | 200 | 0.724 | 0.718 | 0.723 | 0.762 | 0.758 | 0.761 |
|  |  | 500 | 0.754 | 0.754 | 0.754 | 0.788 | 0.788 | 0.788 |
|  |  | 1000 | 0.762 | 0.762 | 0.762 | 0.797 | 0.797 | 0.797 |
|  | Pyramid | 200 | 0.652 | 0.624 | 0.62 | 0.724 | 0.718 | 0.718 |
|  |  | 500 | 0.692 | 0.69 | 0.691 | 0.763 | 0.762 | 0.763 |
|  |  | 1000 | 0.705 | 0.705 | 0.705 | 0.774 | 0.774 | 0.773 |
|  | Diamond | 200 | 0.73 | 0.72 | 0.725 | 0.768 | 0.761 | 0.766 |
|  |  | 500 | 0.759 | 0.757 | 0.758 | 0.795 | 0.794 | 0.795 |
|  |  | 1000 | 0.769 | 0.768 | 0.769 | 0.803 | 0.803 | 0.803 |
| Low | Linear | 200 | 0.55 | 0.468 | 0.451 | 0.535 | 0.421 | 0.418 |
|  |  | 500 | 0.606 | 0.593 | 0.584 | 0.596 | 0.573 | 0.565 |
|  |  | 1000 | 0.633 | 0.628 | 0.627 | 0.626 | 0.622 | 0.618 |
|  | Inverted pyramid | 200 | 0.369 | 0.302 | 0.285 | 0.421 | 0.372 | 0.359 |
|  |  | 500 | 0.426 | 0.411 | 0.401 | 0.501 | 0.489 | 0.475 |
|  |  | 1000 | 0.477 | 0.474 | 0.471 | 0.544 | 0.539 | 0.536 |
|  | Pyramid | 200 | 0.277 | 0.227 | 0.214 | 0.387 | 0.33 | 0.31 |
|  |  | 500 | 0.302 | 0.298 | 0.273 | 0.462 | 0.446 | 0.429 |
|  |  | 1000 | 0.356 | 0.368 | 0.354 | 0.523 | 0.522 | 0.515 |
|  | Diamond | 200 | 0.373 | 0.279 | 0.264 | 0.435 | 0.354 | 0.349 |
|  |  | 500 | 0.416 | 0.404 | 0.384 | 0.509 | 0.495 | 0.48 |
|  |  | 1000 | 0.487 | 0.487 | 0.48 | 0.554 | 0.554 | 0.547 |
